# Evaluating the New TB Antigen-Based Skin Test to Diagnose TB Infection in South Africa

**DOI:** 10.3390/tropicalmed10120338

**Published:** 2025-11-29

**Authors:** Farzana Sathar, Claire du Toit, Violet Chihota, Conor Eastop, Norbert Ndjeka, Katlego Motlhaoleng, Harry Hausler, Matsie Mphahlele, Khilona Radia, Thobani Ntshiqa, Mark Hatherill, Juli Switala, Salome Charalambous, Kavindhran Velen

**Affiliations:** 1 The Aurum Institute, 33 Wrench Road, Johannesburg 1613, South Africa; 2 School of Public Health, University of Witwatersrand, Johannesburg 2193, South Africa; 3 Division of Infectious Diseases, Department of Medicine, Vanderbilt University Medical Centre, Nashville, TN 37232, USA; 4 Foundation for Innovative New Diagnostics, 1202 Geneva, Switzerland; 5 National Department of Health, Pretoria 0187, South Africa; 6 US Centres for Disease Control and Prevention, Pretoria 0001, South Africa; 7 TB HIV Care, Cape Town 8000, South Africa; 8 Antrum Biotech, Cape Town 7925, South Africa; 9 South African Tuberculosis Vaccine Initiative, Institute of Infectious Disease and Department of Pathology, University of Cape Town, Cape Town 6850, South Africa

**Keywords:** tuberculosis preventive treatment, use cases, programmatic implementation

## Abstract

Mycobacterium tuberculosis (TB) antigen-based skin tests, known as TB-specific skin tests (TBSTs), have been recommended by the World Health Organization (WHO) to test for TB infection (TBI). In light of these new recommendations, we conducted a situational analysis and market assessment to evaluate the utility of testing for TBI in general and of the new TBSTs in South Africa. We found the following barriers to acceptability of testing for TBI overall, regardless of the test: the perceived high prevalence of TBI; prior experiences of poor TB preventive treatment (TPT) uptake, which has led to the removal of TBI tests from the current TPT guidelines; and a poor sensitivity of previous TBI tests in people living with HIV (PLHIV). In addition, further barriers to the new TBSTs in particular were as follows: patient level barriers linked to repeat visits; the need for cold chains; and the need for a strong laboratory system, which reduces the need for point-of-care options. TBI testing was thought to be potentially useful to determine the eligibility for TPT in these use cases: healthcare workers, pregnant women living with HIV and prisoners. One other use case was in the TB diagnoses of children, where it was thought that a positive immunological test (TST/IgRA/TBST) could indicate a TB contact and serve as a ‘rule in’ test to strengthen the evidence for TB disease as a cause.

## 1. Introduction

Tuberculosis infection (TBI) is defined as a state of persistent immune response to stimulation by *Mycobacterium tuberculosis* antigens with no evidence of tuberculosis disease (TB) [1]. It is estimated that one third of the world’s population has TBI, the majority of whom are not infectious. Studies have shown that 5–10% of those infected will eventually develop TB over their lifetime [1]. The risk for progression from TBI to disease depends greatly on the individual’s immunological status. Once TBI is suspected, TB preventive treatment (TPT) can be used to prevent a progression to TB.

There is currently no gold standard for identifying TBI. Tuberculin skin tests (TSTs), which use an intradermal purified protein derivative to elicit a reaction (TST), and Interferon-Gamma release assays on blood samples (IgRA) have historically been the main classes of tests available for identifying TBI [1]. The selection of tests among these classes has been challenging for TB programmes, with many inherent trade-offs noted previously [2].

In 2022, the World Health Organization (WHO) recommended the use of TB antigen-based skin tests (TBSTs) to diagnose TBI [3]. TBSTs are administered in a similar way to TSTs. For certain manufacturers, the TBST provides optional consumables to overcome known TBI testing barriers, including a smart patch that indicates where to inject and is linked to a mobile application to assist with result interpretation and recording. When possible, this approach removes the need for a second visit, since the patient can upload a picture of TBST induration for the interpretation and next steps. The disadvantages include the need to wait 48 h for a test result; the need for the image to be read by a healthcare worker; and the requirement of a cold chain [4]. Recent trials have shown similar sensitivities in TBSTs (78%), TSTs (81%) and IGRA (84%) [5]. TBSTs have a similar safety profile to that of TSTs. However, unlike TSTs, there is no cross-reactivity with antibodies in Bacillus Calmette–Guérin (BCG)-vaccinated individuals. Therefore, individuals with TBI who have been BCG-vaccinated can be correctly identified and given appropriate treatment. In 2024, South Africa’s BCG vaccination coverage was 74% [6].

In light of the above-mentioned WHO recommendation and the potential advantages of TBSTs over TSTs in the diagnosis of TBI, we revisited the applicability for testing for TBI and the use of TBSTs to determine their utility in a high-TB-burden country like South Africa. We conducted a situational analysis and market assessment by reviewing the guidelines and the literature on TBI prevalence in different population groups. After reviewing the literature, the authors, representing key stakeholders from the National Department of Health, TB programmes, TB researchers and funders, held discussions to contextualise the findings and explore the potential role of TBSTs within South Africa. We present our findings and recommendations for key use cases, i.e., specific situations in which TBSTs could potentially be used in high-TB-burden countries. Use cases for TBSTs identified in the WHO recommendation include people living with HIV (PLHIV), children under the age of five years, household contacts with TB disease and healthcare workers. In addition to these use cases, we explored potential TBST use in pregnant women, prisoners and the general population of a high-burden country like South Africa.

## 2. TB and TBI Burden in South Africa

South Africa is a high-burden country for drug-susceptible and -resistant TB and TB/HIV co-infection [7]. The first National TB prevalence survey in South Africa included more than 35,000 participants across nine provinces [8]. The estimated TB prevalence was 852 cases per 100,000 people; it was more common in men, people 65 years and older and PLHIV (1734 cases per 100,000). In addition, recent national programme data indicate that approximately 4% of all people diagnosed with TB have multidrug- or rifampicin-resistant TB (MDR/RR-TB) [9]. The overall burden of TBI in the general South African population is unknown; however, studies in specific high-risk groups show a variation in TBI prevalence [10]: children under five (22%) [11], adolescents in schools (50%) [12,13], household contacts of active TB patients (53%) [11], PLHIV (36%) [10] and gold miners (89%) [14]. A study conducted in collaboration with the National Institute for Communicable Disease and the National Department of Health using IGRA found a TBI prevalence of 53% among healthcare workers [15].

## 3. Current South African Guidelines for TPT

In South Africa, those who have had significant TB exposure or who are at high risk (PLHIV, silicosis or healthcare workers) are primarily evaluated for TB disease, and, if TB is excluded, they are offered TPT. TBI tests can be used at the discretion of the healthcare provider to determine TB infection status if needed and if the test is available. The most recent guidelines on TBI treatment state that TBI tests are not a requirement to start TPT [16]. TBI testing is not a specified requirement in the national TB management and screening guidelines for adult high-risk populations [16,17,18]. TBI testing in South Africa is currently limited to testing children under 5 years old to aid in TB diagnosis.

## 4. Barriers to the Buy-In of TBSTs

Most of the stakeholders saw a limited value in testing for TBI in South Africa, especially since the TB disease burden is high, and a TBI test result would not influence patient management. Other barriers to the introduction of TBSTs for the diagnosis of TBI can be summarised as follows: policy barriers include previous campaigns and an experience of poor TPT uptake related to TSTs that has led to the removal of TBI tests from current guidelines—it was felt that the reversal of years of thinking around this would prove to be difficult. Patient-level barriers include transport costs and (for the use of smart technology) the lack of a smartphone or data/internet connectivity. Programme delivery barriers included already over-burdened healthcare workers who may view the TBST as additional work and a strong laboratory system, which facilitates laboratory testing rather than point-of-care options. The reluctance of many stakeholders was supported by the disappointing experience of the roll-out of other point-of-care tools, such as the Determine TB LAM Ag test (Abbott), with many delays in implementation, difficulties in ensuring quality control, problems with supply and forecasting and reporting difficulties.

## 5. Possible Use Cases for TBSTs

We provide a justification, explore each use case briefly and review whether TBSTs would be appropriate for that population. We also summarise the stakeholder perspectives on use cases and recommendations for increasing the evidence (Table 1).

### 5.1. People Living with HIV

PLHIV are at a greater risk of developing TB infection and disease compared to those without HIV [19]. In South Africa, TPT is currently offered to PLHIV regardless of their TBI status [16]. The value of testing for TBI in PLHIV stems from multiple systematic reviews and meta-analyses of trial results that show that the impact of TPT is mostly in those with confirmed TBI [20]. However, a recent large, randomised trial conducted in South Africa showed that TPT was effective among PLHIV regardless of their TBI status [21]. Also, TBI testing may introduce missed opportunities for TPT initiation and barriers to starting TPT, such as waiting for results, the loss of clients who do not return to facilities for the reading or receipt of results and ultimately a reduction in the total number of individuals initiating TPT. Additionally, testing for TBI among PLHIV with advanced disease may yield in false-negative results [22], which may lead to ongoing transmission, morbidity and preventable death. Therefore, at this stage, PLHIV are not recommended as a use case for TBI testing.

### 5.2. Children Under the Age of 5 Years

In South Africa, a high-burden country in which TB is a differential diagnosis for children who present with a variety of clinical pictures but in whom microbiological confirmation is not often possible, a positive immunological test (TST/IgRA/TBST) can serve as a ‘rule in’ test to add to the overall evidence for TB disease as a cause (in conjunction with clinical, radiological and other tests). A negative result, though, is not a ‘rule out test’. BST has the added advantage of less cross-reactivity and false-positives among those with a prior BCG vaccination. Children with decreased immunity (e.g., HIV, malnutrition) face a triple risk: they have an increased risk of infection progressing to disease, they are less likely to have classical clinical, radiological and microbiological features present and they face the highest risk of death if untreated. Because the risk–benefit ratio of over-treating is different in this subgroup, initiating treatment in response to a positive test is more justified.

### 5.3. Household Contacts

The 2023, a United Nations high-level meeting on TB set a global target of providing TPT to 30 million household contacts by 2027 [23]. Household contacts who have been exposed to TB are at risk for TB and recommended for treatment with TBI [24]. Household contacts are also likely to share the same risk factors for TB as their index cases, which include poverty, environmental and living conditions and health determinants such as HIV status, nutrition and access to healthcare [25]. The current TB guidelines do not include the requirement of TBI tests among household contacts before starting TPT [16]. South Africa has recently incorporated Targeted Universal TB Testing (test and treat approach) for all household contacts [16]. Stakeholders felt that testing for TBI is not warranted due to the need for additional home visits and would be resource intensive for the programme and lead to a delay in starting TPT. Another consideration is the perceived discrimination and stigma within the household if TPT was only offered to household members with TBI.

### 5.4. Healthcare Workers

Healthcare workers are known to be at higher risk of TBI and TB disease through continuous occupational exposure [26]. In the past, it was thought that TBI testing in healthcare workers would have no value due to the very high prevalence in this group, which would render the tests unhelpful. However, a recent unpublished South African study indicated that TBI prevalence among healthcare workers is around 50% [15]. Therefore, one could identify persons who would benefit from TPT, and repeated testing may identify those more likely to develop TB in the near future. One would need to consider the logistics and difficulties of implementing such a programme, which would include busy schedules and an apprehension for TB screening in the workplace. We recommend that healthcare workers be considered for TBI testing and further management. Prioritisation based on age categories could be considered, since co-morbidities increase with age, resulting in a higher TBI prevalence.

### 5.5. Pregnant Women

TB disease in pregnancy can result in poor maternal, pregnancy and infant outcomes. It has been suggested that the immunological changes in pregnancy predispose to the development of TB disease and that diagnosis becomes more difficult and is often delayed [27]. A presentation with clinical TB is common in the postpartum period. In South Africa, Isoniazid for TPT has been routinely offered to pregnant women living with HIV, and there is recent controversy with regard to the safety of antenatal Isoniazid [28]. Both TSTs and IGRA have a reduced sensitivity and specificity during pregnancy; the results differ by gestational age at a time since pregnancy outcome. The TBST has not been assessed in pregnant women. The utility of any immune-based testing in pregnancy has not been confirmed.

### 5.6. Prisoners

Prisoners may benefit from testing for TBI and scaling up TPT to prevent transmission and disease in prisons [29,30]. TBI testing would help identify those more likely to benefit. The prevalence of TBI within this population and the feasibility and acceptability, as well as the cost and policy implications of TPT for prisoners who are not living with HIV, would need to be determined. Due to the confined environment, there is a continuous exposure to TB, so issues arise around the frequency of TBI testing. The feasibility of TBSTs with repeat measurements may be improved though, due to the relative ease of follow-up with this type of test.

### 5.7. General Population for TB Elimination

Although the current TB burden in South Africa (and in other high-burden countries) is too high to justify the need for TBI testing, one other indication for the use of TBSTs would be to test general population members to identify those with TBI and to start a population-wide programme of TPT. Though an attractive option to accelerate efforts to achieve TB elimination, practically, we believe that the number of people who would require TPT would be unachievable. It is felt that, for the time being, the focus should be on finding active cases and possibly treating both TB and TBI, alongside disease monitoring. Once we are close to eliminating TB, then we will want to make more discriminant choices to not over-treat.

## 6. Conclusions

Identifying individuals with TBI is critical in the journey towards eliminating TB. However, in South Africa, as in many high-TB-burden countries, it is assumed that a large proportion of the population at risk are infected; therefore, greater effort is placed on initiating TPT. For operational reasons, TBI tests have not been prioritised and have limited reference in the South African TB management guidelines. We conclude that TBI testing using TBSTs could be used to differentiate TB in unwell children presenting at health facilities who do not have a TB contact. Other use cases for TBSTs could include healthcare workers, pregnant women with HIV and prisoners. Since there is compelling evidence that TPT is effective, safe and does not contribute to anti-TB drug resistance, TPT should be promoted.

## Figures and Tables

**Table 1:** Summary of perspectives on use cases of TBSTs and recommendations regarding increasing the evidence for uses (in order of most mentioned to least mentioned).

Use Cases for TBSTs	Recommendations
Healthcare workers (3) * Children (3) * Low-incidence settings (private sector, low-incidence countries) (2) * Prisoners (2) * Immigrants Homeless people People who use drugs Miners Prior to immune-modulating therapy Pregnant women (to avoid over-exposure to medication) Household contacts: HIV-negative or children 5–18 years PLHIV	Economic analysis and modelling (3) * Cost–benefit analysis of TBI testing compared to testing for disease Balance between cost and added benefits of TBSTs compared to PPD Further research: use in pregnant women living with HIV Pilot context research Qualitative research Lessons learnt from other countries Evaluation in South Africa: Diagnostic performance against IGRA

* Number in brackets indicates the number of stakeholders who mentioned this, if >1 person.

## Data Availability

The raw data supporting the conclusions of this article will be made available by the authors on request.

## References

[1] World Health Organization (WHO) (2018). Latent Tuberculosis Infection Updated and Consolidated Guidelines for Programmatic Management.

[2] Muñoz L., Stagg H.R., Abubakar I. (2015). Diagnosis and management of latent tuberculosis infection. Cold Spring Harb Perspect. Med..

[3] WHO (2022). Module 3: Diagnosis WHO Consolidated Guidelines on Tuberculosis Tests for Tuberculosis Infection.

[4] Pai M., Sotgiu G. (2016). Diagnostics for latent TB infection: Incremental, not transformative progress. Eur. Respir. J. Eur. Respir. Soc..

[5] Aggerbeck H., Ruhwald M., Hoff S.T., Tingskov P.N., Hellstrom E., Malahleha M., Siebert M., Gani M., Diacon A., Novelijc Z. (2019). Interaction between C-Tb and PPD given concomitantly in a split-body randomised controlled trial. Int. J. Tuberc. Lung Dis..

[6] World Health Organization (2024). Bacillus Calmette–Guérin (BCG) Vaccination Coverage.

[7] World Health Organization (WHO) (2022). Global Tuberculosis Report 2022.

[8] Ayles H., Mureithi L., Simwinga M. (2022). The State of Tuberculosis in South Africa: What Does the First National Tuberculosis Prevalence Survey Teach Us?. Lancet Infect. Dis..

[9] Department of Health (2023). 2023–2028 National Strategic Plan for HIV|TB|STIs.

[10] Ncayiyana J.R., Bassett J., West N., Westreich D., Musenge E., Emch M., Pettifor A., Hanrahan C.F., Schwartz S.R., Sanne I. (2016). Prevalence of latent tuberculosis infection and predictive factors in an urban informal settlement in Johannesburg, South Africa: A cross-sectional study. BMC Infect. Dis..

[11] MacPherson P., Lebina L., Motsomi K., Bosch Z., Milovanovic M., Ratsela A., Lala S., Variava E., Golub J.E., Webb E.L. (2020). Prevalence and risk factors for latent tuberculosis infection among household contacts of index cases in two South African provinces: Analysis of baseline data from a cluster-randomised trial. PLoS ONE.

[12] Andrews J.R., Morrow C., Walensky R.P., Wood R. (2014). Integrating social contact and environmental data in evaluating tuberculosis transmission in a South African township. J. Infect. Dis..

[13] Mahomed H., Hawkridge T., Verver S., Geiter L., Hatherill M., Abrahams D.-A., Ehrlich R., A Hanekom W., Hussey G.D. (2011). Predictive factors for latent tuberculosis infection among adolescents in a high-burden area in South Africa. Int. J. Tuberc. Lung Dis..

[14] Hanifa Y., Grant A.D., Lewis J., Corbett E.L., Fielding K., Churchyard G. (2009). Prevalence of Latent Tuberculosis Infection among Gold Miners in South Africa. Int. J. Tuberc. Lung Dis..

[15] Mphahlele M., Omar S., Mwansa J., Ntsele P., Ismail F., Charalambous S., Subrayen P., Mvusi L. Latent Tuberculosis Infection Study in South African Hospitals using interferon gamma release assay. Proceedings of the Union World Conference on Lung Health.

[16] South African National Department of Health (2023). National Guidelines on The Treatment of Tuberculosis Infection.

[17] National Department of Health (2014). South Africa National Tuberculosis Management Guidelines.

[18] South African National Department of Health (2022). South African National Department of Health: Implementation Guideline for the Occupational Health Policy for Health Workers in Respect of HIV and TB.

[19] Winter J.R., Adamu A.L., Gupta R.K., Stagg H.R., Delpech V., Abubakar I. (2018). Tuberculosis infection and disease in people living with HIV in countries with low tuberculosis incidence. Int. J. Tuberc. Lung Dis..

[20] Akolo C., Adetifa I., Shepperd S., Volmink J. (2010). Treatment of latent tuberculosis infection in HIV infected persons. Cochrane Database Syst. Rev..

[21] Rangaka M.X., Wilkinson R.J., Boulle A., Glynn J.R., Fielding K., van Cutsem G., A Wilkinson K., Goliath R., Mathee S., Goemaere E. (2014). Isoniazid plus antiretroviral therapy to prevent tuberculosis: A randomised double-blind, placebo-controlled trial. Lancet.

[22] Liu Q., Yang X., Wen J., Tang D., Qi M., He J. (2023). Host factors associated with false negative results in an interferon-γ release assay in adults with active tuberculosis. Heliyon.

[23] Stop TB Partnership (2023). UNHLM 2023 Country Targets.

[24] Velen K., Shingde R.V., Ho J., Fox G.J. (2021). The effectiveness of contact investigation among contacts of tuberculosis patients: A systematic review and meta-analysis. Eur. Respir. J..

[25] Lonnroth K., Castra K.G., Chakaya J.M., Chauhan L.S., Floyd K., Glaziou P., Raviglione M.C. (2010). Tuberculosis control and elimination 2010–50: Cure, care, and social development. Lancet.

[26] Corbett E.L., Muzangwa J., Chaka K., Dauya E., Cheung Y.B., Munyati S.S., Reid A., Hakim J., Chandiwana S., Mason P.R. (2007). Nursing and Community Rates of Mycobacterium Tuberculosis Infection among Students in Harare, Zimbabwe. Clin. Infect. Dis..

[27] Snow K.J., Bekker A., Huang G.K., Graham S.M. (2020). Tuberculosis in pregnant women and neonates: A meta-review of current evidence. Paediatr. Respir Rev..

[28] Gupta A., Montepiedra G., Aaron L., Theron G., McCarthy K., Bradford S., Chipato T., Vhembo T., Stranix-Chibanda L., Onyango-Makumbi C. (2019). Isoniazid Preventive Therapy in HIV-Infected Pregnant and Postpartum Women. N. Engl. J. Med..

[29] Telisinghe L., Charalambous S., Topp S.M., Herce M.E., Hoffmann C.J., Barron P., Schouten E.J., Jahn A., Zachariah R., Harries A.D. (2016). HIV and tuberculosis in prisons in sub-Saharan Africa. Lancet.

[30] Narayan A., Salindri A.D., Keshavjee S., Muyoyeta M., Velen K., Rueda Z.V., Croda J., Charalambous S., García-Basteiro A.L., Shenoi S.V. (2023). Prioritizing persons deprived of liberty in global guidelines for tuberculosis preventive treatment. PLoS Med..

